# Flexible Fibrous Visible Light Sensors Based on Spiropyran for Wearable Devices, Electronic Skins, and Thermal Management Fabrics

**DOI:** 10.1002/smsc.202400018

**Published:** 2024-08-01

**Authors:** Guiqing Dang, Kaifang Chen, Yuncong Luo, Ronghua Hu, Yutao Huang, Henghui Tang, Bingquan Huang, Jinlong Sun, Xi Liu, Yancheng Wu, Longfei Fan, Qinghua Wu, Feng Gan

**Affiliations:** ^1^ College of Textile Science and Engineering Wuyi University Jiangmen Guangdong 529020 China

**Keywords:** conductive fibers, electronic skin, spiropyran, thermal management fabrics, visible light sensors, wearable devices

## Abstract

Visible light is an important energy source for all living organisms on Earth. Given the importance of visible light, visible light sensors have attracted widespread interest from scientists. With the rapid development of wearable devices, the sensors used in them need to be flexible, stretchable, and lightweight. Herein, an intelligent electrolyte based on spiropyran (SP) that responds to visible light is developed. The reversible change rate in the electrical resistance of an SP/FeCl_3_·6H_2_O methyl cyanide (MeCN) aqueous solution under visible light irradiation is as high as 19.26%. Additionally, flexible and conductive fibrous visible light sensors with a core‐sheath structure are prepared using an SP/FeCl_3_·6H_2_O MeCN aqueous solution and silicon rubber hollow fibers as the core and outer layers, respectively. These fibrous visible light sensors are then woven into fabrics with multiple functions, such as sensing and locating visible light, reversible photochromism, and thermal management. The fibrous visible light sensors and fabrics prepared in this study have broad development prospects and application potential in the fields of fashion, smart textiles, flexible conductive fibers, flexible fibrous sensors, electronic skins, and wearable devices.

## Graphical abstract

Flexible fibrous visible light sensors with a core‐sheath structure are prepared using a spiropyran (SP)/FeCl_3_·6H_2_O methyl cyanide aqueous solution and silicon rubber hollow fibers as the core and outer layers. These sensors are woven into fabrics with multiple functions, such as sensing and locating visible light, reversible photochromism, and thermal management.

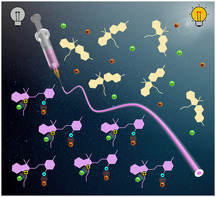

## 1 Introduction

Sunlight is the most basic energy source for the continued existence and development of all living organisms on Earth. Due to the important role of visible light, animals and plants have evolved to sense visible light. Animals perceive visible light with their eyes, using photoreceptor cells to absorb and distinguish photons from different wavelengths to construct an image,^[^ ^1^ ^]^ while plants use photoreceptor proteins, such as phytochromes, cyanobacteriochromes, and phycobiliproteins, to sense different light colors^[^ ^2^ ^]^ and adapt to various environments.

Given the importance of visible light to plants and animals, the development of visible light sensors has attracted substantial attention. Most of the visible light sensors reported to date rely on crystalline semiconductor materials (Table S1, Supporting Information), including thin films (MoS_2_, Cu_2_O, Ga–In–Zn–O (GIZO)/In–Zn–O/GIZO, CdS, WSe_2_, C_3_N_4_, WS_2_, Cu_0.2_Zn_0.8_S, Zn_0.8_Mg_0.2_S, ZnS–Mg, Fe‐doped Bi_2_S_3_, CdS_1−*x*_Se_*x*_, ZnSe, graphene/WS_2_, and CuS), nanoscale materials (CdS, ZnO, GaN, Si nanowires, single‐crystal Se nanobelts, MnO_2_/Co_3_O_4_ with N and S co‐doped graphene oxide hybrid composites, 2D InSe, p‐Si/n–ZnO nanorods and CsPbBr_3_ perovskite quantum dots), and microwires (GaN).^[^ ^3^, ^4^, ^5^, ^6^, ^7^, ^8^, ^9^, ^10^, ^11^, ^12^, ^13^, ^14^, ^15^, ^16^, ^17^, ^18^, ^19^, ^20^, ^21^, ^22^, ^23^, ^24^, ^25^, ^26^, ^27^, ^28^ ^]^ Consequently, visible light sensors have numerous applications, including space communication, mobile phones, industrial quality control, flame monitoring, air quality monitoring, optical imaging, optoelectronic circuits, military surveillance, and wearable devices.^[^ ^29^, ^30^, ^31^, ^32^ ^]^

In this study, we developed a flexible, stretchable, and conductive fibrous visible light sensor using spiropyran (SP) as an intelligent electrolyte. SP and its derivatives are among the most widely studied organic photochromic compounds.^[^ ^33^, ^34^, ^35^, ^36^, ^37^, ^38^, ^39^, ^40^ ^]^ SP demonstrates a reversible conversion from its closed form (SP) to its open form (merocyanine (MC)) in response to different stimuli such as light, solvents, temperature, pH, external electric fields, and stress. As a result, it possesses several remarkable characteristics, including photochromism, solvatochromism, thermochromism, acidochromism, electrochromism, and mechanochromism.^[^ ^41^, ^42^, ^43^, ^44^, ^45^, ^46^ ^]^ As representative molecular switches, the two isomers (SP and MC) have completely different physical and chemical properties. Consequently, SP and its derivatives have been widely used in biological imaging, optical data storage, fluorescent molecular switches, chemical sensors, drug delivery, and controlled wettability applications.^[^ ^47^, ^48^, ^49^, ^50^, ^51^, ^52^ ^]^ When SP is in the open form (MC), its solution color is usually purple, owing to the presence of MC. Because of the phenoxy anions and nitrogen cations of the MC molecules, we speculated that the MC solution is electrically conductive. When the purple MC solution was irradiated with visible light, MC transformed into the closed‐form SP through molecular rearrangement, resulting in a light‐yellow color of the solution. Owing to the disappearance of phenoxy anions and nitrogen cations, the electrical resistance of the SP solution should increase compared to that of the MC solution. Due to the responsiveness of the SP to visible light and the accompanying changes in the electrical resistance, the SP solution should possess excellent visible light sensing capabilities.

To produce flexible fibrous visible light sensors, the prepared SP/metal salt solution with negative photochromism was injected into a silicone rubber hollow fiber to form a core‐sheath multifunctional fiber for smart textiles and wearable devices. As the core layer, the prepared SP/metal salt solution possessed electrical conductivity, visible light sensation, and negative photochromism; while the silicone rubber hollow fiber, as the outer layer, possessed good flexibility and excellent transparency. Due to the fluidity of the SP/metal salt solution within the core layer, the deformation of the outer layer (silicone rubber hollow fiber) did not cut off the SP/metal salt solution in the core layer. As a result, the fabricated fiber retained good electrical conductivity. Based on the above design, a multifunctional visible light sensor with flexibility, stretchability, conductivity, negative photochromism, and visible light sensation was successfully fabricated. The prepared fibrous visible light sensors were further woven into a fabric that could sense visible light and locate its position. It could be seen as an electronic skin with visible light sensing and locating. Owing to its negative photochromism, the prepared fabric could also be seen as an intelligent textile that could change color and adjust its temperature reversibly under exposure to visible light.

## 2 Results and Discussion

The realization of visible light sensing based on the reversible MC‐to‐SP conversion in solution and resulting changes in electrical resistance faces several obstacles. First, common SP and its derivatives typically exhibit positive photochromism in solution, and the closed form (SP) is more stable than the open form, known as MC. Consequently, to utilize the open‐form MC to sense visible light, it is necessary to continuously apply ultraviolet (UV) light to the SP solution to maintain it in an open‐form state (MC). However, because of the need for continuous UV irradiation, it is not suitable for practical applications. Additionally, UV light has a weak penetration ability and can be harmful to organisms. Second, SP and MC solutions have high electrical resistance because SP is a neutral organic compound and MC is a zwitterion. Third, the relative changes of electrical resistance due to the reversible visible‐light‐induced MC‐to‐SP conversion are rather low and therefore unsuitable for visible light sensing.

**Figure 1:**
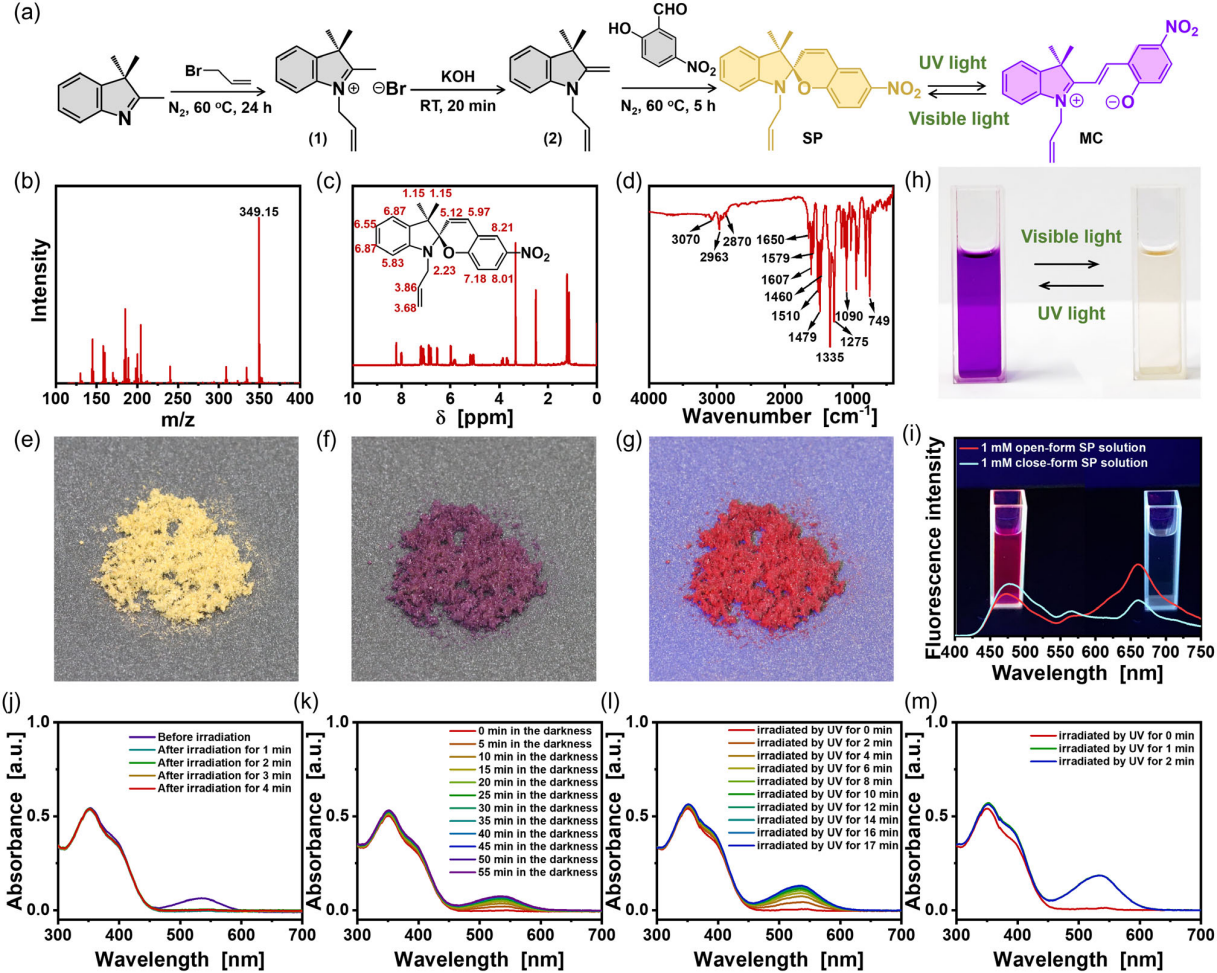
a) Synthesis of SP. b–e) (b) Mass spectrum, (c) ^1^H nuclear magnetic resonance (NMR) spectrum, (d) Fourier transform infrared (FTIR) spectrum, and (e) optical microscopy image of SP. f) Reversible color change of SP powders after irradiation with UV light. g) Fluorescence of SP powders. h) Reversible color change of 1 mM SP MeCN solution after exposure to visible light/UV light. i) Fluorescence spectra of 1 mM SP and MC MeCN aqueous solutions. j) UV–vis spectra of 1 mM open‐form SP MeCN aqueous solution after exposure to visible light (≈54 000 lx). k) UV–vis spectra of 1 mM close‐form SP MeCN aqueous solution in the dark. l,m) UV–vis spectra of 1 mM close‐form SP MeCN aqueous solution after UV light irradiation. (l) Distance between the quartz cell and UV lamp (16 W) is 20 cm. (m) Distance between the quartz cell and UV lamp (60 W) is 2 cm.

To address these problems, we synthesized an SP derivative (**Figure** **1**a) exhibiting positive photochromism in the solid state and negative photochromism in a 60% MeCN aqueous solution. Therefore, it is possible to make its open form (MC) more stable than its closed form (SP) by selecting suitable solvents instead of continuous UV irradiation. To address the issue of inadequate electrical conductivity of the prepared SP or MC solution, an equimolar metal salt was added to the SP solution as an auxiliary electrolyte. This greatly decreases its electrical resistance. To our surprise, adding equimolar metal salts to the SP solution can also solve the problem of low reversible changes in the electrical resistance during the transformation between MC and SP under visible light and darkness.

In the following, the proposed SP is characterized, the importance of metal salts as auxiliary electrolytes is verified, and the factors that affect the functional properties are discussed.

### 2.1 SP Characterization

**Figure 2:**
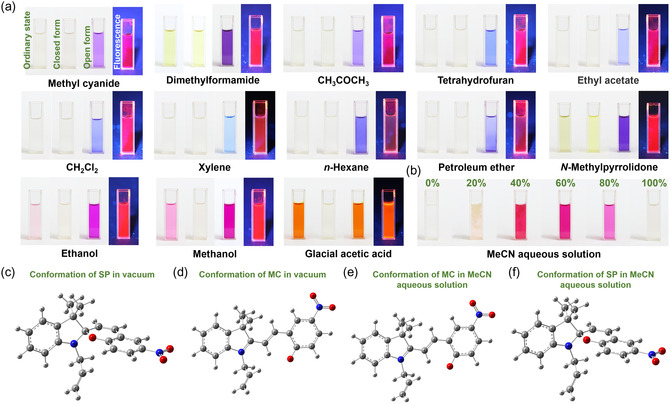
a) Color of 1 mM SP solutions in different organic solvents (left to right: ordinary state, closed form, open form, and fluorescence). b) Color of 1 mM SP solutions in different concentrations of MeCN aqueous solution (MeCN content = 0, 20, 40, 60, 80, and 100 vol%). c,d) Geometry‐optimized structures of (c) SP and (d) MC in vacuum. e,f) Geometry‐optimized structures of (e) SP and (f) in MC 60 vol% MeCN aqueous solution. The gray, red, white, and blue atoms denote C, O, H, and N atoms, respectively.

Figure 1d depicts the FTIR spectrum of synthesized SP. The peaks at 2963 and 2870 cm^−1^ correspond to the stretching vibrations of the carbon–hydrogen bonds (–C—H) of methyl (–CH_3_) of SP. The nitro group (–NO_2_) of SP is confirmed by the peaks at 1510 and 1335 cm^−1^, and the C–N stretching vibration peak is observed at 1275 cm^−1^. The peaks corresponding to the aromatic ring vibration appear at 1607, 1579, 1479, and 1460 cm^−1^. The peaks observed at 1650 cm^−1^ indicate the stretching vibration caused by the C=C bonds in the SP. The peak at 3070 cm^−1^ corresponds to the stretching vibration of carbon–hydrogen bonds of –CH=CH_2_ of the SP structure. The FTIR spectrum confirms that the synthesized product is SP. The molecular structure of the synthesized product can also be confirmed through ^1^H NMR spectroscopy (DMSO, δ ppm, Figure 1c) and mass spectrum results (Figure 1b). The peak at *m*/*z* = 349.15 corresponds to the [M + H]^+^ species. Based on the analysis of the above experimental results, it can be concluded that the synthesized yellow powders are the target product SP. Optical microscopy images of the synthesized SP and its solution are given in Figure 1e,h. Upon UV irradiation, the synthesized SP powders change from yellow to purple (Figure 1f) and produce bright purple‐red fluorescence (Figure 1g). The purple powders returned to yellow after visible light irradiation. It is evident that the prepared SP, similar to other reported positive SPs, transforms from the closed form (yellow powders, Figure 1e) to the open form (purple powders, Figure 1f) under UV irradiation, and that the SP is more stable than the MC.^[^ ^33^, ^34^, ^35^, ^36^, ^37^, ^38^, ^39^, ^40^ ^]^ To verify this assumption, the Gaussian 16 suite of programs was utilized to optimize the structure and Gibbs free energies (*G*) of the closed‐form isomer (SP) and open‐form isomer (MC) of synthesized SP under vacuum.^[^ ^53^ ^]^ Based on the results of theoretical calculations (**Figure** **2**c,d), the Gibbs free energies of SP and MC were −1147.448505 Hartree and −1147.440818 Hartree, respectively. Consequently, it is evident that Δ*G* = *G*_SP_−*G*_MC_ = −1147.448505 Hartree − (−1147.440818 Hartree) = −0.0077 Hartree = −19.75 KJ mol^−1^, indicating that the closed‐form SP (SP) is more stable than the open form (MC).

When the synthesized yellow SP powders are dissolved in different organic solvents (MeCN, dimethylformamide (DMF), CH_3_COCH_3_, tetrahydrofuran (THF), ethyl acetate, CH_2_Cl_2_, xylene, n‐hexane, petroleum ether, N‐methylpyrrolidone, ethanol (EtOH), methanol (MeOH), and glacial acetic acid), the color of the prepared solutions are shown in Figure 2a. The color of the prepared mixed solutions changes from light yellow or colorless when the SP in the solution is in the closed form as a result of visible light irradiation to blue, purple, or orange when the SP in the solution is in the open form as a result of UV light irradiation (Figure 2a). Therefore, it is possible to infer whether SP is open or closed in different organic solutions based on the color of the mixed solution. Most ordinary states of different SP/organic solutions are light yellow or colorless, except for the SP/EtOH (light pink), SP/MeOH (pink), and SP/glacial acetic acid (orange) solutions. Based on analysis of these experimental results, when SP is dissolved in EtOH or MeOH, a few SP molecules are in the open form and the other SP molecules are in the closed form, resulting in light pink and pink mixed solutions, respectively. When the SP is dissolved in glacial acetic acid, the color of the prepared solution is the same as that when the SP molecules are completely opened in the solution through UV light irradiation. It is evident that when SP is dissolved in glacial acetic acid, all SP molecules exist in the open form. Moreover, it is evident that the SP molecules are partially or completely in the open form in EtOH, MeOH, and glacial acetic acid due to the ability of the selected organic solvent to dissociate hydrogen ions. When the selected organic solvent can dissociate hydrogen ions more easily, more SP molecules transform from closed to open form. To test this hypothesis, we selected water, which can also dissociate hydrogen ions, as the solvent. However, because the prepared SP cannot dissolve in water, we chose another organic solution, MeCN, which cannot dissociate hydrogen ions, and mixed it with water to investigate the color change of SP at different concentrations of the MeCN aqueous solution (Figure 2b). When pure water is used as the solvent, the obtained solution is colorless because the SP is insoluble in water. When pure MeCN is used as the solvent, the mixed solution is colorless because the SP molecules are in the closed form in MeCN. When the selected solvent is the MeCN aqueous solution, as the concentration of MeCN increases from 20% to 60%, the color of the SP MeCN aqueous solution gradually changes to purple, indicating that an increasing number of SP molecules transform from the closed form to the open form. When an 80% MeCN aqueous solution is selected, the mixed solution's color changes from purple (60% MeCN aqueous solution) to light purple (80% MeCN aqueous solution), which is caused by a decrease in the proportion of water. The color change is due to the introduction of water, which proves that the dissociation of hydrogen ions of the selected solvent can promote the transition of SP molecules from closed to open. As shown in Figure 2b, incomplete dissolution of the SP powder can still be observed in the 20% and 40% MeCN aqueous solutions, whereas the SP powder has been completely dissolved in the 60% and 80% MeCN aqueous solutions. However, considering the subsequent introduction of various metal salts as auxiliary electrolytes into the SP solution, a 60% MeCN aqueous solution with higher water content is selected as the optimal SP solvent. Glacial acetic acid is not used as a solvent as it is considerably more corrosive than the MeCN aqueous solution. Therefore, unless otherwise stated, the 60% MeCN aqueous solution was selected for SP in this work. When 60% MeCN aqueous solution was used as the solvent, as shown in Figure 2e,f, Δ*G* = *G*_SP _− *G*_MC_ =−1147.490685Hartree − (−1147.496185Hartree) = 0.0055Hartree = 14.44 KJ mol^−1^, indicating the open‐form MC to be more stable. Therefore, the SP molecules in the 60% MeCN aqueous solution are open, leading to the purple color of the SP MeCN aqueous solution.

Irradiating the SP MeCN aqueous solution with UV light produces intense purple‐red fluorescence (Figure 1i). After exposure to visible light, the SP in the MeCN aqueous solution closes molecularly through molecular arrangement. When exposed to UV light, no fluorescence can be observed (Figure 1i). After being left in the dark, the colorless SP solution turns purple, indicating molecular rearrangement of SP into MC.

UV–vis spectral analysis was used to characterize the negative reversible photochromism of the produced SP MeCN aqueous solution. Figure 1j displays the time‐dependent absorption spectra of 0.05 mM SP MeCN aqueous solution under visible light (≈54 000 lx). As shown in Figure 1j, there is an obvious peak that appears at 450–600 nm at 0 min, and maximum absorption at 535 nm. This peak suggests open‐form SP molecules. There are no peaks that appear at 450–600 nm as time increases to 1 min, and the mixed solution turns from purple to colorless, showing that SP molecules in the MeCN aqueous solution are closed. When the colorless SP MeCN aqueous solution is placed in darkness, the closed‐form SP eventually becomes open‐form MC (Figure 1k). Over time, the peak at 535 nm gradually intensifies. The peak at 450–600 nm reverts to its original position after 50 min. Thus, after 50 min in darkness, the closed‐form SP molecules have entirely changed into the open‐form MC. As seen above, the produced SP is stable in the open form in the MeCN aqueous solution under dark conditions but quickly closes after visible light irradiation. The above results reveal the reversibility of the SP–MC transition.

The closed‐form SP molecules can also quickly transform to the open form under UV light stimulation (Figure 1l). The obtained colorless SP solution was irradiated with a UV lamp (16 W), and the characteristic peak of SP at 450–600 nm gradually increased with increasing time. After 16 min, the height of this peak did not continue to increase. Therefore, we believe that ultraviolet light can significantly shorten the time for SP to transition from open form to closed form, from 50 min under dark conditions to 16 min under UV light. By selecting a UV lamp with higher power (60 W), the time was further reduced to 1 min (Figure 1m).

### 2.2 SP Utilized as an Intelligent Electrolyte

**Figure 3:**
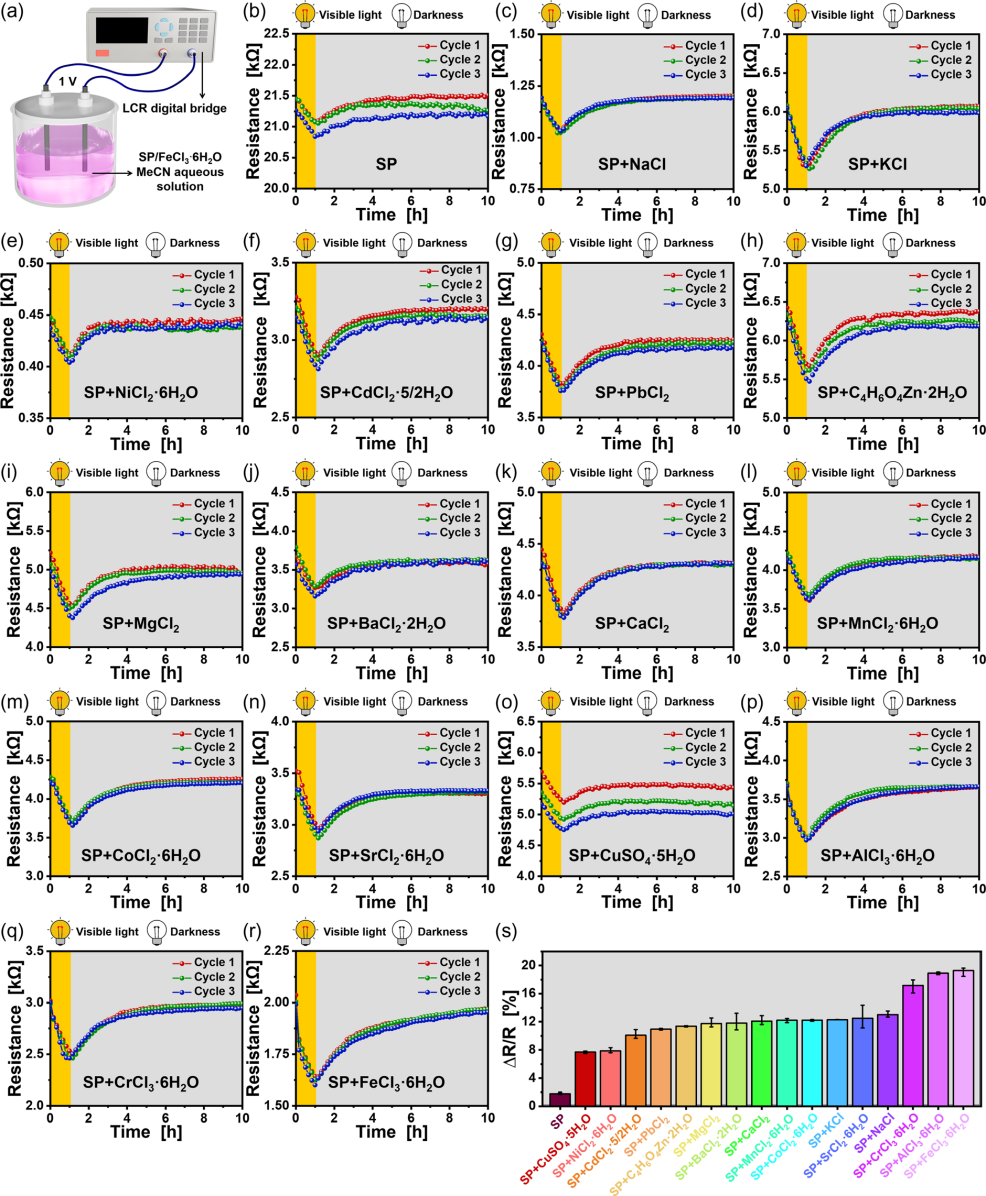
a) Setup used for SP MeCN aqueous solution electrical resistance measurements. b–r) Electrical resistance and s) average reversible change in the electrical resistance of SP/different metal salt (0.5, 0.5 mM) MeCN aqueous solution during three visible light irradiation (1 h) and darkness (9 h) cycles.

The prepared SP MeCN aqueous solution (1 mM) was placed in an electrolytic cell and subjected to electrical resistance measurements using an inductance capacitance resistance (LCR) digital bridge (**Figure** **3**a). The electrical resistance of the SP MeCN aqueous solution is 21.39 kΩ, proving that the prepared SP MeCN aqueous solution indeed is electrically conductive. Upon exposure to visible light for 1 h, its electrical resistance decreases from 21.39 to 21.01 kΩ (Figure 3b). After being placed in the dark for 9 h, the resistance of the solution gradually recovered (Figure 3b). These results demonstrate that the prepared SP MeCN aqueous solution can detect visible light.

There are three types of ions in the SP MeCN aqueous solution: MC zwitterions, a few hydrogen ions, and hydroxide ions produced by water electrolysis. However, owing to the electrostatic attraction of phenoxy anions (on MC molecules) to hydrogen ions, the moving speed of hydrogen ions in the solution is greatly reduced; the nitrogen cations (on MC molecules) also generate electrostatic attraction to the hydroxide ions generated by water electrolysis, thus reducing the moving speed of the hydroxide ions in the solution. The electrical resistance of the SP solution measured by the LCR digital bridge at this point is 21.39 kΩ (Figure 3b). After exposure to visible light, the MC zwitterions transform into nonionic SP molecules. Because of the disappearance of phenoxy anions and nitrogen cations, their electrostatic attraction to hydrogen and hydroxide ions also disappears. Consequently, the moving speed of hydrogen ions and hydroxide ions in the solution recovers, decreasing in the SP solution's electrical resistance to 21.01 kΩ (Figure 3b).

According to Figure 3b, the SP solution's electrical resistance is relatively high (21.39 kΩ), and the electrical resistance change rate before and after visible light irradiation is just 1.78%. To decrease the SP solution's electrical resistance, equimolar metal salts (NaCl, Zn(CH_3_COO)_2_·2H_2_O, AlCl_3_·6H_2_O, KCl, CrCl_3_.6H_2_O, CoCl_2_·6H_2_O, CdCl_2_·5_/2_H_2_O, FeCl_3_·6H_2_O, SrCl_2_·6H_2_O, CuSO_4_·5H_2_O, BaCl_2_·2H_2_O or MnCl_2_·4H_2_O) could be introduced as auxiliary electrolytes into the SP solution. As auxiliary electrolytes, metal salts provide two vital functions in solutions. 1) They decrease the SP solution's electrical resistance; and 2) They increase the reversible change in electrical resistance of the SP solution before and after visible light exposure. The electrical resistance of the SP MeCN aqueous solution decreased significantly after adding equimolar metal compounds to increase the ion concentration. The electrical resistance of the mixed solution containing metal salts averages 3.84 kΩ (Figure 3c–r). This result is considerably less than the electrical resistance of 21.39 kΩ in the SP solution.

The movement speed of metal ions and acid radicals dissociated from various metal salts in the mixed solution was substantially influenced by the zwitterionic MC molecules present in the SP/metal salt MeCN aqueous solution via electrostatic attraction. After exposure to the visible light, the MC molecules in the mixed solution transform into SP molecules. This transition eliminates the electrostatic attraction between phenoxy anions/nitrogen cations and metal ions/acid radicals, restoring their movement speed of metal ions and acid radicals. Moreover, the SP/metal salt MeCN aqueous solution experiences a substantial reduction in electrical resistance. Reintroducing the SP/metal salt MeCN aqueous solution to darkness increases its electrical resistance. The average reversible change in the electrical resistance of the SP/metal salt MeCN aqueous solution under the cyclic stimulation of visible light and darkness is 12.58% (Figure 3s), which is significantly improved compared with that of the SP MeCN aqueous solution (1.78%). The mixed solution's electrical resistance changes the most when FeCl_3_·6H_2_O is used as the metal salt, averaging 19.26% (Figure 3s). To illustrate the function of SP, the variation in electrical resistance of various metal salt solutions in response to visible light stimulation and darkness was quantified (Figure S2, Supporting Information). As illustrated in Figure S2, Supporting Information, metal salt solution resistance remains unaltered when exposed to visible light. Hence, we believe that SP causes the SP/metal salt MeCN aqueous solution's reversible electrical resistance to change when stimulated with visible light or darkened.

FTIR spectra (Figure S3, Supporting Information) and mass spectra (Figure S4, Supporting Information) were employed to ascertain whether the selected metal ions coordinate with the MC molecules. The addition of metal salts did not change the positions of existing FTIR peaks or induce the emergence of new ones. Moreover, no molecular ion peaks corresponding to metal coordination were observed in the mass spectra. Based on the experimental findings, the metal ions cannot coordinate with the open‐form SP.

In our previous study,^[^ ^53^ ^]^ we used MC diol MC(OH)_2_ as an intelligent electrolyte. However, MC(OH)_2_ and SP have completely different chemical structures, stimulus responsiveness, and mechanisms underlying reversible negative photochromism. The SP/FeCl_3_·6H_2_O solution in aqueous MeCN prepared herein underwent a higher reversible resistance change and exhibited more diverse stimulus responsiveness than MC(OH)_2_, i.e., SP was concluded to be a better choice for intelligent electrolyte fabrication than MC(OH)_2_.

### 2.3 Flexible Fibrous Visible Light Sensors

**Figure 4:**
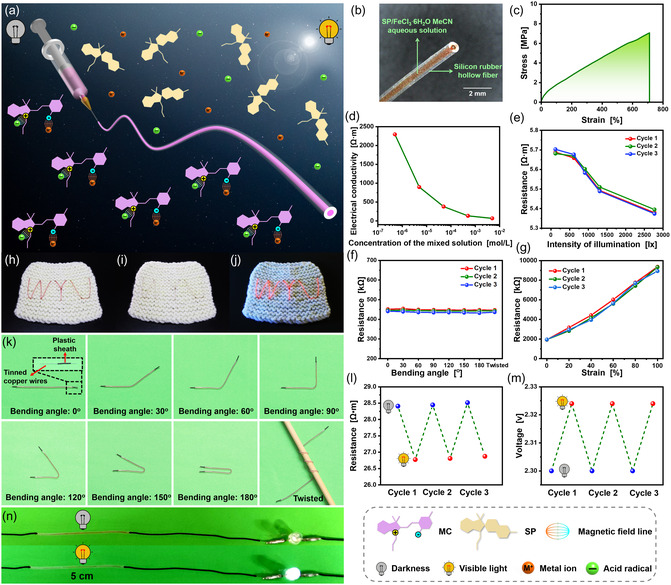
a–c) (a) Conceptual design, (b) optical microscopy image, and (c) typical tensile stress–strain curve of the flexible fibrous visible light sensor. d–g) The electrical resistance of the fibrous visible light sensor as a function of (d) the concentration of the mixed solution, (e) the intensity of the visible light, (f) bending angle, and (g) strain. h–j) The patterns embroidered on the fabric with the flexible fibrous visible light sensors under (h) darkness, (i) visible light, and (j) UV light. k) Images of the prepared fibrous visible light sensor bent to different shapes. l) Electrical resistance of the prepared fibrous visible light sensor during three visible light irradiation (1 min) and darkness (50 min) cycles. m) Voltage applied to the LED bulb. n) Brightness of the LED bulb.

Flexible fibrous visible light sensors were prepared by injecting an SP/FeCl_3_·6H_2_O MeCN aqueous solution (5 mM) into silicone rubber hollow fibers (**Figure** **4**a). This fibrous visible light sensor has a core‐sheath structure. The internal SP/FeCl_3_·6H_2_O MeCN aqueous solution possesses the ability to conduct electricity and sense visible light; while the external silicone rubber hollow fiber provides sufficient flexibility, good mechanical properties (Figure 4c), and excellent transparency (Figure S5, Supporting Information). Figure 4b,k shows the digital photos of the prepared fibrous visible light sensor. Owing to the yellow color of the FeCl_3_·6H_2_O MeCN aqueous solution, the SP/FeCl_3_·6H_2_O MeCN aqueous solution is purple‐red instead of purple. Increased concentration of the SP/FeCl_3_·6H_2_O mixed solution significantly affects the electrical resistance of the produced fibrous visible light sensor. The higher the concentration, the lower the electrical resistance of the fibers (Figure 4d). Figure 4d shows that the electrical resistance of the fibrous visible light sensor is 68.82 Ω m at a concentration of 0.01 m for the SP/FeCl_3_·6H_2_O MeCN aqueous solution. Although the mixed solution concentration is reduced to 0.00001 m, it maintains a good conductivity of 898.44 Ω m (Figure 4d). According to Figure 4e, the electrical resistance of the produced visible light sensor decreases with the enhancement of visible light intensity.

The electrical resistance of the fabricated fibrous visible light sensor was tested and recorded upon bending (Figure 4f,k) and extending (Figure 4g) due to its flexibility. According to Figure 4f, when the fibrous visible light sensor was bent, the electrical resistance of the sensor under bending (30°, 60°, 90°, 120°, 150°, and 180°, Figure 4k) or twisting (Figure 4k) conditions remains almost unchanged (Figure 4f) because the cross‐sectional area of the solution does not significantly change during bending or twisting process. However, when the prepared visible light sensor is stretched, the cross‐sectional area of the solution within the fiber decreases with increasing fiber strain during elongation, resulting in an increase in the electrical resistance of the prepared visible light sensor with increasing strain (Figure 4g). Given that sensor resistance was affected by visible light irradiation and stretching but not bending, we concluded that the developed sensor should not be stretched but can be bent. In addition, the produced fibrous visible light sensors could be embroidered onto the surface of textiles to form various patterns (Figure 4h–j), which can be used in wearable devices. The embroidered patterns also exhibit photochromism (Figure 4h,i) and fluorescence (Figure 4j), making them suitable for use in the fashion and military fields.

An adjustable direct current (DC) power source, light‐emitting diode (LED) bulb, and the prepared fibrous visible light sensor were connected in series. Under dark conditions, the DC power supply was adjusted to distribute a voltage of 2.30 V to the LED bulb, which exactly reached the turn‐on voltage of the LED bulb (2.30 V). At this point, the LED bulb emitted a faint glow (Figure 4n); when visible light was used to irradiate the fibrous visible light sensor, it caused a decrease in its electrical resistance (Figure 4l), resulting in an increase in the voltage distributed to the LED bulb from 2.30 to 2.32 V (Figure 4m), leading to a further increase in brightness of the LED bulb (Figure 4n). Moreover, the color of the selected fibrous visible light sensor underwent a transition from purple‐red to yellow. Consequently, the presence of visible light in the vicinity of the fibrous visible light sensor can be determined by assessing the luminosity of the LED bulb.

**Figure 5:**
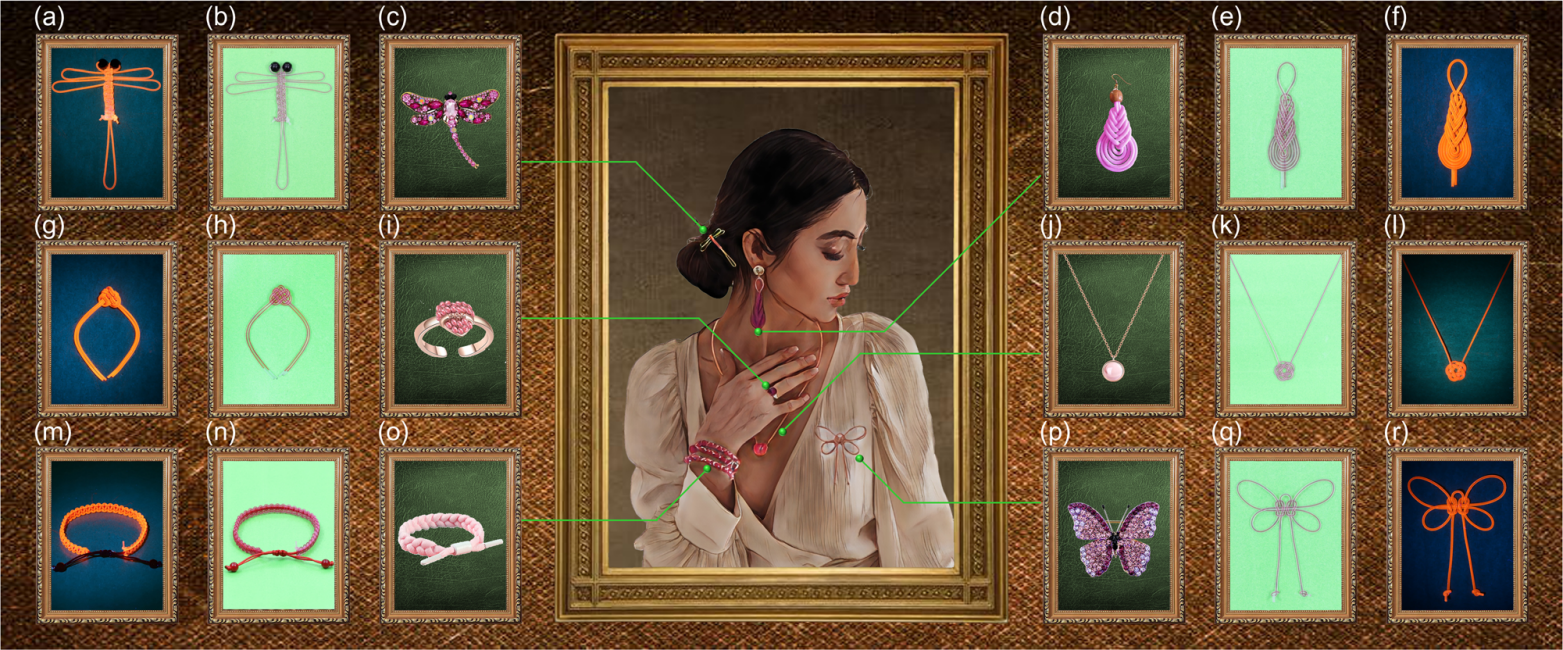
a,f,g,l,m,r) Digital photograph of the fluorescence of (a) a hair clip, (f) an earring, (g) a ring, (l) a necklace, (m) a bracelet, and (f) a brooch. b,e,h,k,n,q) (b) A hair clip, (e) an earring, (h) a ring, (k) a necklace, (n) a bracelet, and (q) a brooch made of the prepared fibrous visible light sensors using embroidery. c,d,i,j,o,p) Digital photograph of (c) a hair clip, (d) an earring, (i) a ring, (j) a necklace, (o) a bracelet, and (p) a brooch.

Additionally, the prepared fibrous visible light sensors were also utilized to prepare various decorative accessories, such as hair clips, rings, bracelets, earrings, necklaces, and brooches (**Figure** **5**), through weaving. These accessories not only could sense visible light but also exhibited aesthetically appealing fluorescence upon UV irradiation. Thus, the developed visible light sensors were concluded to have broad development prospects in the fields of fashion and wearable devices.

### 2.4 Electronic Skin for Sensing and Locating Visible Light

**Figure 6:**
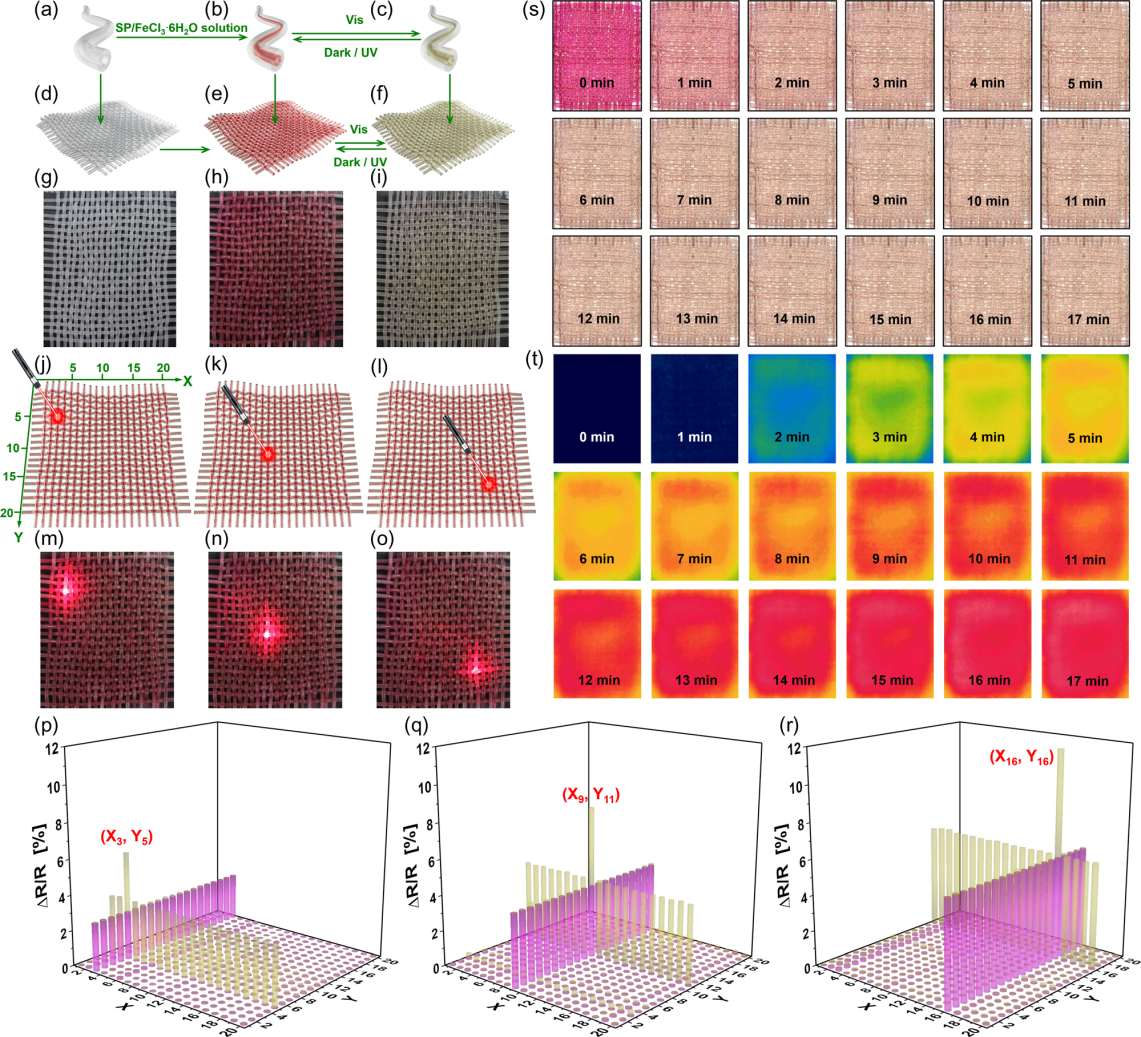
a–c) Structures of the (a) representative silicone rubber hollow fiber, (b) prepared fibrous visible light sensor in the dark, and (c) prepared fibrous visible light sensor upon visible light irradiation. d–i) Structures of (d,g) the fabric made of silicon rubber hollow fibers, (e,h) the fabric made of the prepared fibrous visible light sensors under dark conditions, and (f,i) the fabric made of the prepared fibrous visible light sensors under visible light irradiation. j–o) A laser pointer was used to irradiate different positions (j,m) (*X*_3_, *Y*_5_), (k,n) (*X*_9_,*Y*_11_), and (l,o) (*X*_16_, *Y*_16_) of the prepared fabric. s,t) (s) Color and (t) temperature changes of the prepared fabric under visible light irradiation. p–r) The sum of the change rate in electrical resistance at each intersection of the two fibers when visible light irradiates the position of (p) (*X*_3_, *Y*_5_), (q) (*X*_9_, *Y*_11_), and (r) (*X*_16_, *Y*_16_). The purple columns/yellow columns represent the absolute value of the change rate of electrical resistance of fibers parallel to the horizontal direction (*X*_1_–*X*_20_)/perpendicular to the horizontal direction (*Y*_1_–*Y*_20_), respectively.

The prepared fibrous visible light sensors were further woven into textiles (**Figure** **6**a–f). Because all the fibrous visible light sensors in this fabric can sense visible light and exhibit photochromism (Figure 6a–c), the resulting fabric also exhibits these two functions. Therefore, under visible light irradiation, the prepared fabric can quickly transform from its original purple red to light yellow (Figure 6d–i), making it suitable for use in smart textiles.

Because of the fixed position of each fiber in the fabric, the positions of each fiber parallel to the horizontal direction can be defined as *X*_1_ to *X*_20_ (Figure 6j), and the positions of each fiber perpendicular to the horizontal direction can be defined as *Y*_1_ to *Y*_20_ (Figure 6j). If not all fibers of the prepared fabric are stimulated by visible light radiation, but rather by localized visible light radiation, the electrical resistance of the irradiated fibers will change due to partially visible light stimulation. The greater the change in the electrical resistance of the fibers, the stronger the visible light radiation. Moreover, because the area irradiated by visible light includes fibers parallel or perpendicular to the horizontal direction, the position of the visible light can be determined by the positions of these fibers, which have the largest change in electrical resistance.

A laser pointer was used to locally irradiate the fabric with visible light to verify this assumption (Figure 6j–o). The coordinates of visible light are (*X*_3_, *Y*_5_) (Figure 6j,m), (*X*_9_, *Y*_11_) (Figure 6k,n), and (*X*_16_, *Y*_16_) (Figure 6l,o). An LCR digital bridge was used to test the change rate of electrical resistance (Δ*R*/*R*) of each fiber on the fabric. The purple and yellow columns in Figure 6p–r represent the absolute value of changes in the electrical resistance of fibers parallel to the horizontal direction (*X*_1_–*X*_20_) and perpendicular to the horizontal direction (*Y*_1_–*Y*_20_), respectively. It is evident that the intersection of the two fibers with the largest sum of change rate of electrical resistance is the location of visible light irradiation. Based on Figure 6p–r, the positions with the largest sum of absolute values of the electrical resistance change rates are (*X*_3_, *Y*_5_), (*X*_9_, *Y*_11_), and (*X*_16_, *Y*_16_), respectively. The experimental results are consistent with the position of the visible light provided by the laser pointer. Consequently, the above results prove that the prepared fabric not only exhibits the ability to sense visible light and photochromism but also can locate the position of visible light, enabling it to be used as an electronic skin to sense and locate visible light.

### 2.5 Thermal Management Fabrics

Given that the resulting fabric exhibited reversible photochromism upon visible light irradiation, we concluded that the color change could be used in smart textiles, and cause temperature change under visible light irradiation. As shown in Figure 6s, the fabric changes from purple‐red to light yellow within 1 min under visible light irradiation. After 15 min of visible light irradiation (≈126 000 lx), the surface temperature of the fabric increased from 25.8 to 43.2 °C (Figure 6t). A control fabric without reversible photochromism was prepared using dye solutions of similar color instead of the SP/FeCl_3_·6H_2_O MeCN aqueous solution, as shown in Figure S6, Supporting Information. After 15 min of visible light irradiation, the temperature increased from 26.1 to 47.5 °C. Based on the analysis of these results, the surface temperature of the prepared fabric was ≈4 °C lower than that of the control under the same experimental conditions.

## 3 Conclusion

In this research, a SP compound was synthesized and utilized as an intelligent electrolyte. The synthesized SP exhibited positive photochromism in the solid state and negative photochromism in MeCN aqueous solution. The prepared SP/metal salt mixed solution could sense visible light, and the change rate of the electrical resistance of the SP/FeCl_3_·6H_2_O MeCN aqueous solution was as high as 19.26%. We further developed a flexible fibrous visible light sensor featuring a core‐sheath structure by utilizing the stimulus‐response of SP/FeCl_3_·6H_2_O MeCN aqueous solution to visible light. Additionally, we prepared a series of wearable decorative accessories. The prepared flexible fibrous visible light sensors were further woven into textiles that could sense and locate visible light. The prepared fabric could also be used as an electronic skin. Additionally, the prepared fabric exhibited photochromism, so it could intelligently adjust its surface temperature when exposed to visible light. Therefore, it could be regarded as a smart textile for thermal management and could be applied in intelligent garments and military fields.

## 4 Experimental Section

4.1

4.1.1

##### Materials

2‐Bromopropene, 2,3,3‐trimethylindolenine, 2‐hydroxy‐5‐nitrobenzaldehyde, potassium hydroxide, methyl tert‐butyl ether, ethanol, ethyl acetate, n‐hexane, dimethyl sulfoxide, acetone, acetonitrile, xylene, N,N‐dimethylformamide, petroleum ether, methanol, tetrahydrofuran, dichloromethane, NaCl, Zn(CH_3_COO)_2_·2H_2_O, AlCl_3_·6H_2_O, KCl, CrCl_3_.6H_2_O, CoCl_2_·6H_2_O, CdCl_2_·5_/2_H_2_O, FeCl_3_·6H_2_O, SrCl_2_·6H_2_O, CuSO_4_·5H_2_O, BaCl_2_·2H_2_O, MnCl_2_·4H_2_O, and Na_2_SO_4_ were procured from Macklin, China. All the reagents and materials were procured from commercial suppliers and utilized without undergoing additional purification unless otherwise specified.

##### Synthesis of 5‐ Allyl −1‐(2‐Hydroxyethyl)‐2,3,3‐Trimethyl‐3H‐Indolium Bromide (1) (Figure 1a)

In 20 mL of acetonitrile, 2.42 g (20 mmol) 2‐bromopropene and 2.55 g (16 mmol) 2,3,3‐trimethylindolenine were dissolved. Under N_2_, the temperature of the mixture was increased to 60 °C for 24 h. After removing residual 2‐bromopropene and acetonitrile via rotary evaporation, the product underwent three washes with n‐hexane. As a result, a purple‐red powder (2.84 g) was obtained, representing a yield of 66.71%. The calculated mass of [M‐Br]^+^ C_14_H_18_NBr, indicated by the mass spectrum, is 200.1, while the observed mass is 200.14 (Figure S1a, Supporting Information). Proton nuclear magnetic resonance (^1^H NMR, 400 MHz, dimethyl sulfoxide‐d6, Figure S1b, Supporting Information): *δ* (ppm) 7.93, 7.59, 7.37, 6.11, 5.44, 5.22, 2.88, 1.57, and 1.37. Figure S1c, Supporting Information, depicts the FTIR spectrum of the resultant product (KBr/cm^−1^: 2980, 2870, 1630, 1610, 1590, 1480, and 1460).

##### Synthesis of 1‐Allyl‐3,3‐Dimethyl‐2‐Methyleneindoline (2) (Figure 1a)

In water, 2.79 g of compound 1 (10.00 mmol) and 1.68 g of KOH (30.00 mmol) were dissolved. 20 min were spent stirring the mixture at ambient temperature. The mixture was subsequently extracted using 3 × 20 mL of methyl tert‐butyl ether. A dark red oily liquid product was obtained through evaporation of the combined organic layer desiccated over Na_2_SO_4_, yielding 80.19%. The mass spectrum indicates that the precise mass of [M + H]^+^ C_14_H_18_N is 200, whereas the observed mass is 200.14 (Figure S1d, Supporting Information). ^1^H NMR (400 MHz, dimethyl sulfoxide‐d6, Figure S1e, Supporting Information): *δ* (ppm) 7.27, 6.70, 5.78, 5.08, 4.14, 3.89, 3.08, 2.20, 1.27 and 1.11. Figure S1f, Supporting Information, depicts the FTIR spectrum of compound 2 (KBr/cm^−1^: 2960, 2870, 1650, 1610, 1580, 1490, and 1460).

##### Synthesis of 1'‐Allyl‐3',3'‐Dimethyl‐6‐Nitrospiro[chromene‐2,2'‐Indoline] (SP) (Figure 1a)

In 20 mL of acetonitrile, 0.98 g (4.90 mmol) compound 2 and 0.68 g (4.10 mmol) 2‐hydroxy‐5‐nitrobenzaldehyde were dissolved and stirred at 60 °C for 5 h while being surrounded by N_2_. The acetonitrile was eliminated by rotary evaporation. The prepared product was recrystallized 3 times in MeCN aqueous solution (v:v = 7:3) and a yield of 71.74% was achieved for a yellow powder weighing 1.34 g. The mass spectrum indicates that the precise mass of [M + H]^+^ C_21_H_20_O_3_N_2_ is 349; the observed mass is 349.15 (Figure 1b). Figure 1c illustrates the ^1^H NMR (400 MHz, dimethyl sulfoxide‐d6) spectrum: *δ* (ppm) 8.21, 8.01, 7.18, 6.87, 6.55, 5.97, 5.83, 5.12, 3.86, 3.68, 2.23, and 1.15. Figure 1d illustrates the FTIR spectrum of the resultant product (KBr/cm^−1^: 3070, 2963, 2870, 1650, 1607, 1579, 1510, 1479, 1460, 1335, 12 751 090, and 749).

##### Preparation of the Flexible Fibrous Visible Light Sensor

SP/FeCl_3_·6H_2_O MeCN aqueous solution was prepared by adding 10 mL of a 0.01 m SP MeCN aqueous solution (v:v = 6:4) and 10 mL of a 0.01 M FeCl_3_·6H_2_O MeCN aqueous solution (v:v = 6:4) to a flask and stirring at ambient temperature for 5 min. A conductive, flexible, visible light sensor was created by injecting the prepared SP/FeCl_3_·6H_2_O MeCN aqueous solution into a hollow silicone rubber fiber (1.0 mm external diameter, 0.5 mm internal diameter).

##### Characterization

Mass spectra were taken using a Thermo Fisher mass spectrometer. FTIR spectra were recorded using a Nicolet Is50 spectrometer (Thermo, US). ^1^H NMR spectra were measured by an AVANCE III (400 MHz, Bruker, Germany). Absorption spectra were measured with a UV‐6100 UV–vis spectrophotometer (Shanghai Yuanxi Instruments, China). Japanese Shimadzu RF‐6000 spectrophotometer acquired fluorescence spectra (excitation wavelength: 430 nm). UV light irradiation was carried out with UV lamps (16 W, Shanghai Yixin Scientific Instrument Co., Ltd, China; 60 W, Ningbo Patti E‐commerce Co., Ltd, China). The surface morphologies of the prepared fibrous visible light sensor were recorded using a Japanese KEYENCE VHX‐6000 optical microscopy. The surface temperature of the prepared fabric was recorded using a Uti 320E thermal imager (UNI‐T Co., Ltd, China). Visible light irradiation was carried out using a white‐light LED lamp (18 W, Eryuehe Lighting Co., Ltd., China) with a dominant wavelength, peak wavelength, and color temperature of 476.0, 451.4, and 8082 K, respectively. Theoretical calculations were performed via the Gaussian 16 suite of programs.^[^ ^54^ ^]^

##### Statistical Analysis

Quantitative data were described in the form of means ± standard deviation, such as the error bars in the figures. At least three cycles were conducted to test the average reversible change rate in the electrical resistance. All statistical analyses were carried out with the Origin software package.

## Conflict of Interest

The authors declare no conflict of interest.

## Supporting information

Supplementary Material

## Data Availability

The data that support the findings of this study are available from the corresponding author upon reasonable request.
