# Supplementary material for: Flexible Fibrous Visible Light Sensors Based on Spiropyran for Wearable Devices, Electronic Skins, and Thermal Management Fabrics
Source: Small Sci. 2024 Aug 1;4(9):2400018. doi: 10.1002/smsc.202400018 (PMC11934970; doi:10.1002/smsc.202400018)
Supplement: Supplementary file 1 — Supplementary Material [file SMSC-4-2400018-s001.pdf]

Supporting Information

Flexible fibrous visible light sensors based on spiropyran for wearable devices, electronic skins and thermal management fabrics

Guiqing Dang, Kaifang Chen, Yuncong Luo, Ronghua Hu, Yutao Huang, Henghui Tang, Bingquan Huang, Jinlong Sun, Xi Liu, Yancheng Wu, Longfei Fan, Qinghua Wu*, and Feng Gan**

Table S1. Typical visible light sensors

Materials	Classification		Form			Ref.
	Inorganic materials	Organic materials	Thin films	Nanoscale materials	Microwires	
MoS_2	●[a)]	○[b)]	●	○	○	1
WSe_2	●	○	●	○	○	2
Silicon	●	○	●	○	○	3
Selenium nanobelt	●	○	○	●	○	4
Cu_2O	●	○	●	○	○	5
Ga–In–Zn–O (GIZO)/In–Zn–O(IZO)/GIZO	●	○	●	○	○	6
GaN	●	○	○	○	●	7
CdS	●	○	○	●	○	8
CdS	●	○	●	○	○	9
MnO_2/Co_3O_4 with N and S co-doped graphene oxide hybrid composite	●	○	○	●	○	10
MoS_2	●	○	●	○	○	11
InSe	●	○	○	●	○	12
C_3N_4	●	○	●	○	○	13
ZnO	●	○	○	●	○	14
WS_2	●	○	●	○	○	15
WS_2	●	○	●	○	○	16
$Cu_{0.2}Zn_{0.8}S$	●	○	●	○	○	17
$Zn_{0.8}Mg_{0.2}S$	●	○	●	○	○	18
GaN	●	○	○	●	○	19
ZnS-Mg	●	○	●	○	○	20
p-Si/n-ZnO	●	○	○	●	○	21
Fe-doped Bi_2S_3	●	○	●	○	○	22
CdS	●	○	●	○	○	23
Silicon	●	○	○	●	○	24
$CdS_{1-x}Se_x$	●	○	●	○	○	25
CdS	●	○	○	●	○	26
ZnSe	●	○	●	○	○	27
CdS	●	○	○	●	○	28
Graphene/ WS_2	●	○	●	○	○	29
$CsPbBr_3$	●	○	○	●	○	30
CuS	●	○	●	○	○	31

[a)]●: Applicable. [b)]○: Not applicable.

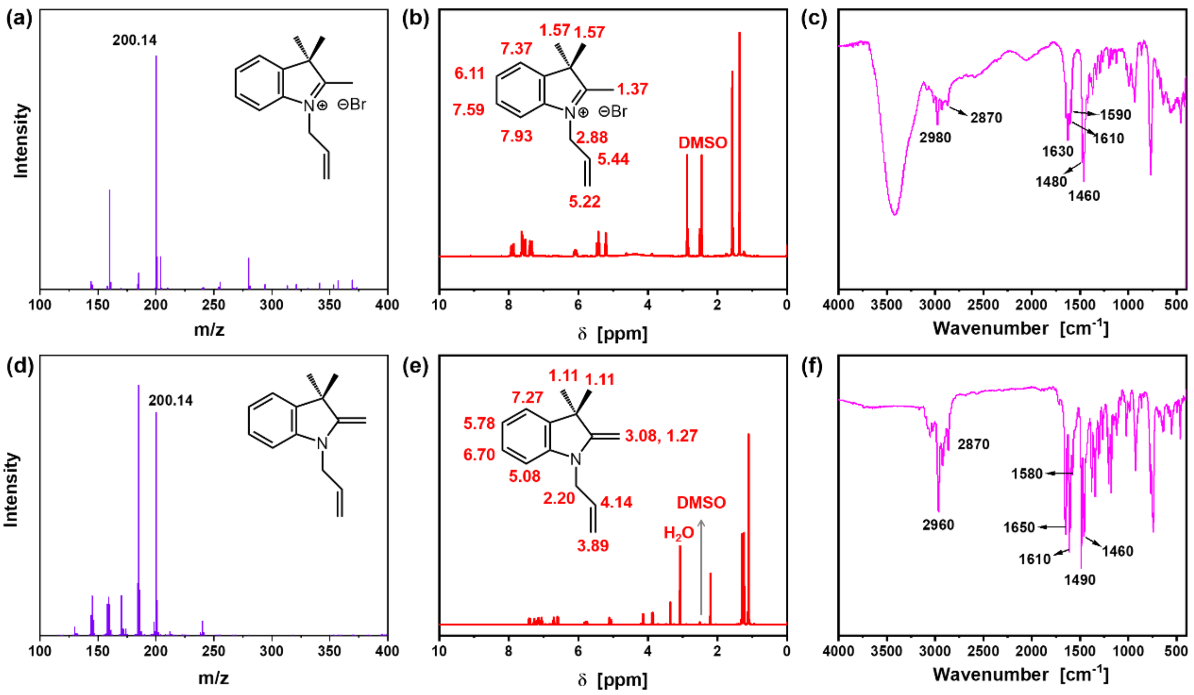

Figure S1. (a~c) (a) Mass spectrum, (b) ^{1}H NMR spectrum and (c) FTIR spectrum of compound 1; (d~f) (d) Mass spectrum, (e) ^{1}H NMR spectrum and (f) FTIR spectrum of compound 2.

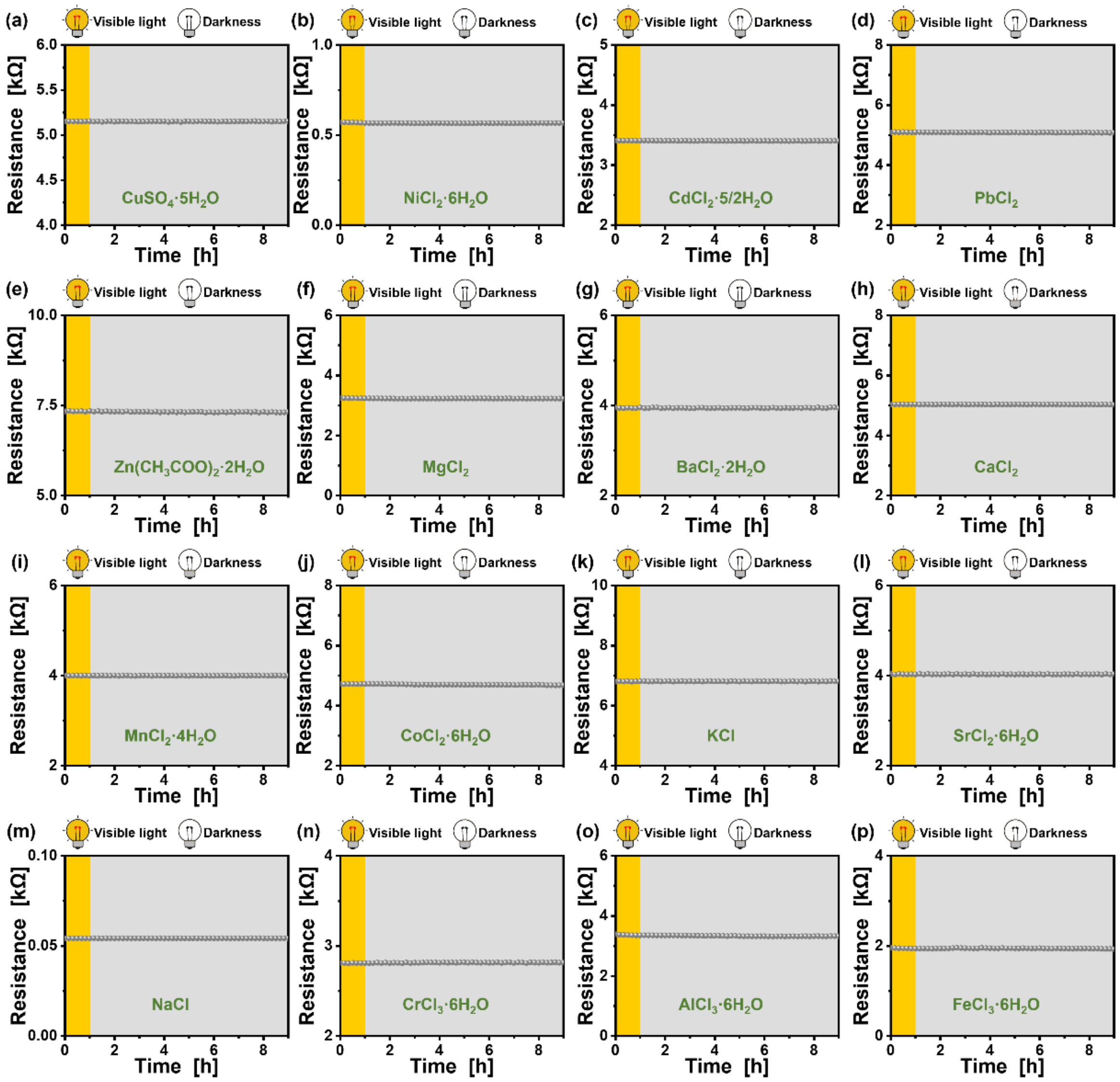

Figure S2. (a-p) Electrical resistance of metal salts (0.5 mM) dissolved in the MeCN aqueous solution (v/v=6:4) during visible light irradiation (1 h) and darkness (9 h): (a) $CuSO_4·5H_2O$ solution; (b) $NiCl_2·6H_2O$ solution; (c) $CdCl_2·{}^5/_2H_2O$ solution; (d) $PbCl_2$ solution; (e) $Zn(CH_3COO)_2·2H_2O$ solution; (f) $MgCl_2$ solution; (g) $BaCl_2·2H_2O$ solution; (h) $CaCl_2$ solution; (i) $MnCl_2·4H_2O$ solution; (j) $CoCl_2·6H_2O$ solution; (k) KCl solution; (l) $SrCl_2·6H_2O$ solution; (m) NaCl solution; (n) $CrCl_3.6H_2O$ solution; (o) $AlCl_3·6H_2O$ solution; (p) $FeCl_3·6H_2O$ solution.

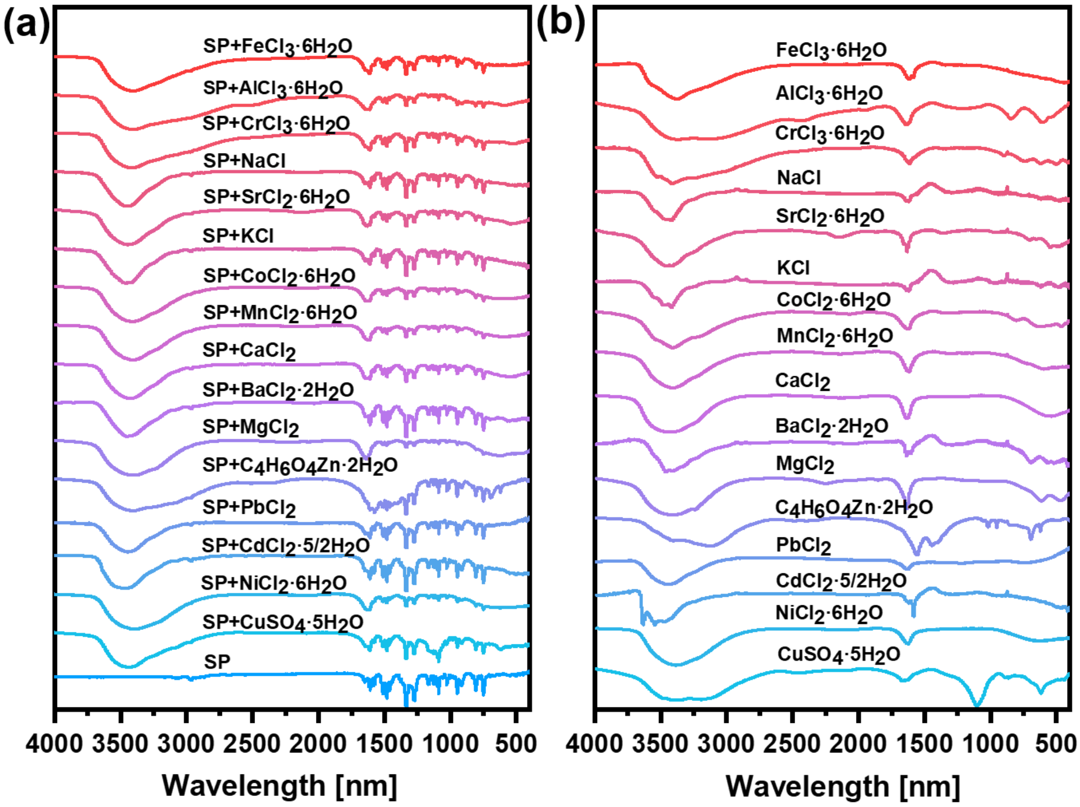

Figure S3. FTIR spectra of (a) SP/metal salt and (b) pure metal salts.

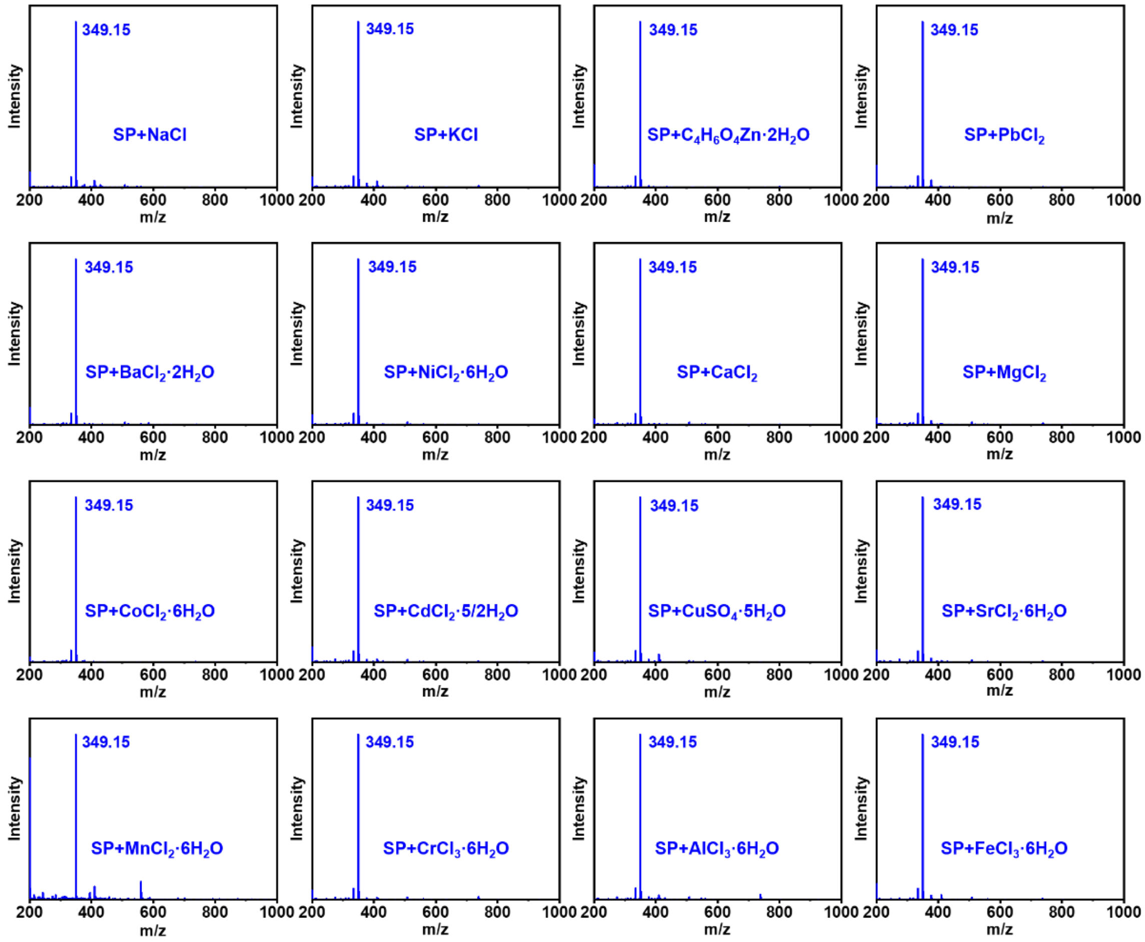

Figure S4. MS spectra of SP/metal salt (0.5 mM, 0.5 mM) dissolved in the MeCN aqueous (v/v=6:4) solution.

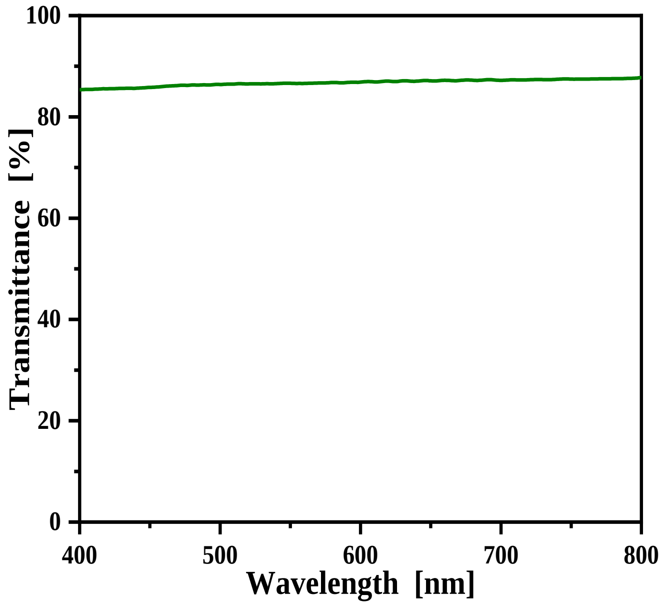

Figure S5. VIS spectra of silicon rubber hollow fiber.

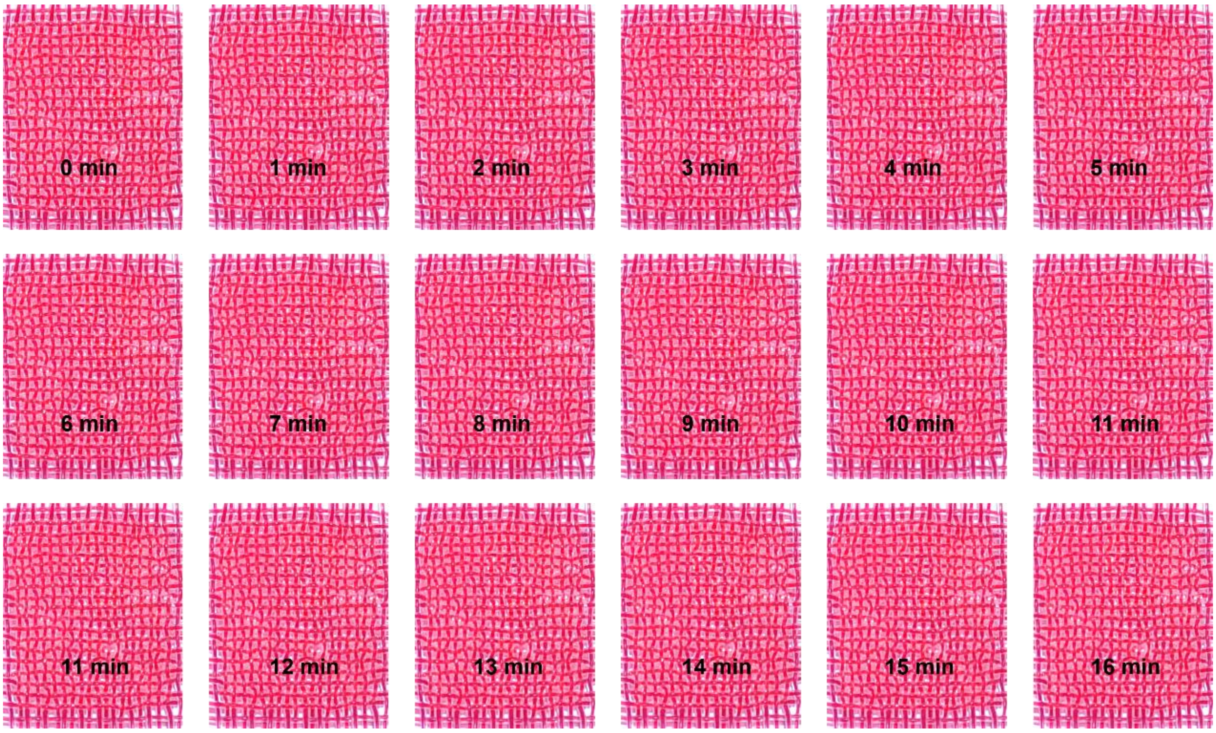

Figure S6. Color change of the control fabric under visible light irradiation.

Supplementary references

[S1] N. Perea-López, Z. Lin, N. R. Pradhan, A. Iñiguez-Rábago, A. L. Elías, A. McCreary, J. Lou, P. M. Ajayan, H. Terrones, L. Balicas, M. Terrones, *2D Mater.* **2014**, *1*, 011004.

[S2] Y. Li, X. Feng, M. Sivan, J. F. Leong, B. Tang, X. Wang, J. N. Tey, J. Wei, K. W. Ang, A. V. Y. Thean, *IEEE Sens. J.* **2020**, *20*, 4653.

[S3] Y. Huang, C. Tsou, *IEEE Photon. Technol. Lett.* **2021**, *33*, 217.

[S4] Y. Niu, A. Qin, W. Song, M. Wang, X. Gu, Y. Zhang, M. Yu, X. Zhao, M. Dai, L. Yan, Z. Li, Y. Fan, *J. Nanomater.* **2012**, *2012*, e384671.

[S5] A. M. Selman, M. A. Mahdi, Z. Hassan, *Physica E Low Dimens. Syst. Nanostruct.* **2017**, *94*, 132.

[S6] S. Jeon, S.-E. Ahn, I. Song, C. J. Kim, U.-I. Chung, E. Lee, I. Yoo, A. Nathan, S. Lee, K. Ghaffarzadeh, J. Robertson, K. Kim, *Nat. Mater.* **2012**, *11*, 301.

[S7] W. Song, X. Wang, C. Xia, R. Wang, L. Zhao, D. Guo, H. Chen, J. Xiao, S. Su, S. Li, *Nano Energy* **2017**, *33*, 272.

[S8] J.-H. Im, H.-R. Kim, B.-G. An, Y. W. Chang, M.-J. Kang, T.-G. Lee, J. G. Son, J.-G. Park, J.-C. Pyun, *Biosens. Bioelectron.* **2017**, *92*, 221.

[S9] I. M. S. Mohammed, G. M. M. Gubari, M. E. Sonawane, R. R. Kasar, S. A. Patil, M. K. Mishra, V. V. Kutwade, R. Sharma, *Appl. Phys. A* **2021**, *127*, 597.

[S10] K. Adaikalam, S. Ramesh, P. Santhoshkumar, H. S. Kim, H.-C. Park, H.-S. Kim, *Int. J. Energy Res.* **2022**, *46*, 4494.

[S11] Y. Tong, Y. Liu, D. S. H. Chan, J. Thong, C. Zhu, *Sens. Actuator A Phys.* **2017**, *266*, 205.

[S12] Z. Jin, F. Ye, X. Zhang, S. Jia, L. Dong, S. Lei, R. Vajtai, J. T. Robinson, J. Lou, P. M. Ajayan, *ACS Nano* **2018**, *12*, 12571.

[S13] R. K. Gupta, A. A. Al-Ghamdi, F. El-Tantawy, W. A. Farooq, F. Yakuphanoglu, *Mater. Lett.* **2014**, *134*, 149.

[S14] A. Takshi, J. Bebeau, F. Rahimi, presented at SPIE Organic Sensors and Bioelectronics IX, San Diego, 9, **2016**.

[S15] N. Perea-López, A. L. Elías, A. Berkdemir, A. Castro-Beltran, H. R. Gutiérrez, S. Feng, R. Lv, T. Hayashi, F. López-Urías, S. Ghosh, B. Muchharla, S. Talapatra, H. Terrones, M. Terrones, *Adv. Funct. Mater.* **2013**, *23*, 5511.

[S16] R. Canton-Vitoria, S. Nufer, X. Che, Y. Sayed-Ahmad-Baraza, R. Arenal, C. Bittencourt, A. Brunton, A. B. Dalton, C. P. Ewels, N. Tagmatarchis, *Mater. Adv.* **2020**, *1*, 2459.

[S17] G. M. M. Gubari, S. M. I. Mohammed, N. P. Huse, A. S. Dive, R. Sharma, *J. Electron. Mater.* **2018**, *47*, 6128.

[S18] A. S. Dive, N. P. Huse, K. P. Gattu, R. Sharma, *Sens. Actuator A Phys.* **2017**, *266*, 36.

[S19] S. Han, S. Noh, J.-W. Kim, C.-R. Lee, S.-K. Lee, J. S. Kim, *ACS Appl. Mater. Interfaces* **2021**, *13*, 22728.

[S20] A. S. Dive, N. P. Huse, K. P. Gattu, R. B. Birajdar, D. R. Upadhyay, R. Sharma, *J. Mater. Sci. Mater. Electron.* **2017**, *28*, 15161.

[S21] S. Thongma, K. Tantisantisom, N. Grisdanurak, T. Boonkoom, *Sens. Actuator A Phys.* **2019**, *296*, 324.

[S22] S. Rajeswari, M. M. Ibrahim, I. L. P. Raj, J. Hakami, M. Imran, S. AlFaify, M. Shkir, *Sens. Actuator A Phys.* **2022**, *345*, 113759.

[S23] S. R. Gosavi, C. P. Nikam, A. R. Shelke, A. M. Patil, S.-W. Ryu, J. S. Bhat, N. G. Deshpande, *Mater. Chem. Phys.* **2015**, *160*, 244.

[S24] A. A. Abdul-Hameed, M. A. Mahdi, B. Ali, A. M. Selman, H. F. Al-Taay, P. Jennings, W.-J. Lee, *Superlattices Microstruct.* **2018**, *116*, 27.

[S25] F. Y. Siddiqui, S. U. Shaikh, D. J. Desale, D. S. Upadhye, S. V. Mahajan, A. V. Ghule, P. Varshney, S.-H. Han, R. Sharma, *Mater. Sci. Semicond. Process.* **2014**, *27*, 404.

[S26] B.-G. An, H.-R. Kim, M.-J. Kang, J.-G. Park, Y. W. Chang, J.-C. Pyun, *Anal. Chim. Acta* **2016**, *927*, 99.

[S27] H. K. Sadekar, A. V. Ghule, R. Sharma, *Compos. B Eng.* **2013**, 44, 553.

[S28] S. U. Shaikh, D. J. Desale, F. Y. Siddiqui, A. Ghosh, R. B. Birajadar, A. V. Ghule, R. Sharma, *Mater. Res. Bull.* **2012**, *47*, 3440.

[S29] T. Leng, K. Parvez, K. Pan, J. Ali, D. McManus, K. S. Novoselov, C. Casiraghi, Z. Hu, *2D Mater.* **2020**, *7*, 024004.

[S30] H.-R. Kim, J.-H. Bong, J.-H. Park, Z. Song, M.-J. Kang, D. H. Son, J.-C. Pyun, *ACS Appl. Mater. Interfaces* **2021**, *13*, 29392.

[S31] N. P. Huse, A. S. Dive, K. P. Gattu, R. Sharma, *Mat. Sci. Semicon. Proc.* **2017**, *67*, 62.
